# Association between plasma leptin/adiponectin ratios with the extent and severity of coronary artery disease

**DOI:** 10.1186/s12872-020-01723-7

**Published:** 2020-11-04

**Authors:** Asghar Rahmani, Yaser Toloueitabar, Yousef Mohsenzadeh, Roholla Hemmati, Kourosh Sayehmiri, Khairollah Asadollahi

**Affiliations:** 1 School of Medicine, Ilam University of Medical Science, Ilam, Iran; 2 Rajaie Cardiovascular Medical and Research Center, Iran University of Medical Sciences, Tehran, Iran; 3 Department of Cardiology, Faculty of Medicine, Ilam University of Medical Science, Ilam, Iran; 4 Department of Biostatistics, Faculty of Health, Ilam University of Medical Sciences, Ilam, Iran; 5 Department of Social Medicine, Faculty of Medicine, Ilam University of Medical Science, Ilam, Iran

**Keywords:** Coronary artery disease, Leptin, Adiponectin, Leptin/adiponectin ratio, Gensini score

## Abstract

**Background:**

Leptin can have a direct effect on endothelial and vascular smooth muscle cells and high level of leptin is involved in the pathogenesis of atherosclerosis. This study aimed to determine the relationship between leptin/adiponectin (L/A) ratio and the extent and severity of coronary artery disease (CAD).

**Methods:**

This case-control study was conducted in an educational hospital in Ilam, Iran from June 2014 to September 2015. Totally 300 participants including 150 patients with CAD (case group) and 150 healthy individuals (control group) were selected and their plasma leptin, adiponectin and leptin/adiponectin ratio was measured. The extent and severity of coronary artery disease were assayed based on the number of involved vessels and Gensini score (GS) and the relation between scores and L/A findings were compared between cases and controls.

**Results:**

Totally, 300 participants including 150 (42.7% male), mean age 59.5 ± 11.4 years as cases and 150 (50.7% male), mean age 59.8 ± 10.7 as controls were analyzed. Plasma level of leptin and L/A ratio were higher in cases compared to controls, but level of adiponectin was significantly lower in CAD patients than the control group. More number of involved coronary vessels was significantly correlated to higher level of plasma leptin, L/A ratio and lower level of adiponectin among case group. Moreover, adiponectin was negatively and leptin or L/A ratio were positively correlated with number of involved vessels. 7.3% of cases had only one involved vessel, 42.7% had two involved vessels, and 50% of total patients had involved vessels and the mean ± SD of GS in the case group was 23.6 ± 6.9.

**Conclusions:**

Plasma levels of leptin, and adiponectin can indicate the extent of coronary artery diseases but leptin may be a better marker of extent of CAD than either L/A ratio or adiponectin separately.

## Background

Coronary artery disease is one of the most common causes of mortality and morbidity worldwide [1]. Despite the high prevalence of this disease, the exact mechanisms associated with the development of atherosclerosis remain unclear [2]. Several risk factors associated with the progression of coronary artery disease including obesity, smoking, hypertension, hyperlipidemia and other chronic inflammatory conditions have been identified [3]. Studies suggest that obesity is the one of the most important risk factors for CAD and one of the important factors underlying the link between obesity and CAD is the leptin and adiponectin levels secreted by adipose tissue [4]. Although the main role of leptin is human energy homeostasis and body weight regulation by the brain [5], it also has a variety of functions, such as participation in inflammatory processes, immune function [6], angiogenesis and vascular functions [7]. In addition, leptin can have a direct effect on endothelial and vascular smooth muscle cells [8] and a high level of leptin is involved in the pathogenesis of atherosclerosis [9]. Some reports have shown a significant independent association between leptin and coronary artery calcification (CAC) [10] and the intima-media thickness of common carotid artery in patients with type 2 diabetes mellitus [11]. Though some studies have shown that leptin is an independent risk factor for CHD [12]; however, studies on leptin and CAD have also reported conflicting results [13]. Adiponectin is known as a cardioprotective and anti-atherogenic mediator [14] and its plasma levels are reduced in obese and dyslipidemic individuals [15]. Some studies have reported correlations between hypoadiponectinemia and an increased risk of CAD [16]. Despite extensive studies, the pro-atherogenic effects of leptin and the anti-atherogenic effects of adiponectin in atherosclerotic patients have not been fully defined. The plasma L/A ratio may be a more appropriate indicator for predicting the risk of CAD compared to leptin or adiponectin serum level alone [17, 18]. However, the relationship between L/A ratio and the extent and severity of coronary artery disease is not clear. The aim of this study was to investigate the relationship between the leptin and L/A ratio indices and the severity and extent of coronary artery disease using angiography and GS.

## Methods

### Study design

This case-control study was conducted in patients referred to Mustafa Khomeini Hospital, an educational hospital and major referral medical center, in Ilam, Iran between June 2014 and September 2015. During the mentioned period, all patients with inclusion criteria were selected as cases and for each case a healthy individual at the same date was randomly selected as control. A total of 150 CAD patients underwent coronary angiography in Cardiology Department (case group) and 150 healthy individuals from the same center (control group) were selected as the study subjects. Controls did not show any coronary involvements based on angiographic results. Inclusion criteria for participants were as follow: typical chest pain, coronary involvement after non-invasive evaluations such as physical exam or scan, and post-myocardial infarction (MI), and exclusion criteria were as follow: heart failure (left ventricular ejection fraction < 40%), history of acute myocardial infarction, history of coronary artery diseases by coronary angioplasty, acute or chronic renal failure, hepatic diseases such as nonalcoholic fatty liver disease (NAFLD) or cirrhosis, chronic infection, malignancy, any autoimmune diseases or hematological disorders and any inflammatory or metabolic diseases. To eliminate the effect of glucose and lipid metabolism, patients with diabetes were excluded from the study. Using a standardized questionnaire, the demographic characteristics of participants, including age, sex, cardiovascular risk factors such as smoking, family history of heart diseases, and hypertension (HTN), were obtained. A complete physical examination was performed, and blood pressure (BP) was taken from each patient’s right arm, after 10 min resting in a quiet room, in a seating position. Two successive BP readings were obtained, at 5 min intervals, and their average was considered as patient’s blood pressure. Hypertension was defined as systolic blood pressure (SBP) ≥ 140 mmHg or a diastolic blood pressure (DBP) ≥ 90 mmHg or the use of antihypertensive drugs [19]. The weight and height of participants were measured using a digital scale and tapeline by the same individual. Body mass index (BMI, kg /m^2^) was calculated as the weight (kg) divided by height (m^2^). Waist circumference (WC) was measured at the end of expiration at the narrowest point of the abdomen, approximately 5.2 cm between the umbilicus and hip. This measurement was performed at the maximum bulge point in a standing position, with closed feet.

### Coronary angiography

Coronary angiography was carried out using the standard Judkins technique and a semi-quantitative coronary angiographic system [20]. The angiographic results were collected by two cardiologists, and based on the different findings, the patients were divided into 3 categories. The first category was determined according to the number of involved vessels indicating 1, 2, or 3 involved vessels groups; the second category was determined according to the type of involved vessels indicating the place of involved vessels such as left anterior descending artery (LAD), right coronary artery (RCA), left circumflex artery (LCX), and the diagonal or marginal artery; and the third category was determined according to the rate of stenosis of the vessels indicating a percentage of stenosis such as less than 50%, 50–75% and more than 75%. In this study, coronary artery stenosis was assayed using Gensini scores, which represents both the severity and the extent of coronary atherosclerosis [21]. Gensini score is a widely used mean of quantifying angiographic atherosclerosis, where a zero score indicates absence of atherosclerotic disease. The Gensini score accounts for the degree of artery narrowing as well as locations of narrowing. The severity of stenosis is indicated by the reduction in lumen diameter, and a nonlinear score is assigned to each lesion based upon it.

The studied patients were divided into the following six groups according to the American Heart Association protocols: Score 1—stenosis < 25%; Score 2—stenosis 26–50%; Score 4—stenosis 51–75%; Score 8—stenosis 76–90%; Score 16—stenosis 91–99%; and Score 32—stenosis 100%.

Another grouping of patients was based on the severity of coronary artery lesions using GS as normal coronary artery (Score 0–1), mild lesions (Score 2–20), moderate lesions (Score 20–40), and severe lesions (Score > 40).

### Biochemical assay

After a 12-h fasting, 10 ml/l of vein blood sample was obtained from each patient and after centrifuging by 1000 g for 10 min, and the samples were stored at − 70 °C. Lipid profiles, including triglycerides (TGs), cholesterol (Chol), low density lipoprotein cholesterol (LDL-C) and high density lipoprotein cholesterol (HDL-C), and plasma glucose were measured using routine laboratory techniques (Pars Azmoon kit, Catalog No 1500017, Tehran, Iran for TG and Chol and Zist Shimi kit, Serial No D00749, Tehran, Iran for HDL-C respectively). LDL-C was estimated based on direct measurement with autoanalyzer. ELISA kits were used to assay the ratios of serum adiponectin (R&D Systems, USA) and serum leptin (D&G Diagnostics, Germany), and the assays were conducted according to the manufacturer’s protocol.

### Statistical analysis

Data were expressed as the mean ± SD for quantitative variables and/or frequencies for qualitative variables. The normality of data was assessed using the Kolmogorov-Smirnov test. Student’s t-test and the chi-square tests were applied appropriately. Pearson correlation coefficient method was applied for the correlation between plasma leptin, adiponectin or the L/A ratios and the demographic or clinical characteristics of participants. In addition, Spearman correlation coefficient method was applied for variables without normal distribution. Logistic regression and multivariate analysis were applied for testing the predicting value of some plasma factors for CAD extension and severity. *P* value equal or less than 0.05 was considered statistically significant for all variables. The data analysis was performed via IBM SPSS Statistics for Windows, version 2019 (IBM Corp., Armonk, N.Y., USA). The Epanechnikov Kernel Smoothing was applied to show a non- linear association between adiponectin or leptin and Gensini score.

## Results

All demographic, laboratory, anthropometric and angiographic findings of participants are indicated in Table 1. The male percentage and mean ± SD for age among control and case groups were 50.7%; 59.8 ± 10.7 and 42.7%; 59.5 ± 11.4 years respectively and there were no significant differences for gender or age between groups (*p* = 0.101 and *p* = 0.802, respectively). The angiographic evaluations of patients showed that 7.3% of cases had only one involved vessel, 42.7% had two involved vessels, and 50% of total patients had involved vessels and the mean ± SD of Gensini score in the case group was 23.6 ± 6.9.

**Table 1:** Demographic and clinical characteristics of study participants

Variables	Control group ***n*** = 150	Case group ***n*** = 150	***P*** value
**Demographic data**			
**Sex, Male, n (%)**	76 (50.66)	64 (42.66)	0.101
**Age (years)**	59.78 ± 10.65	59.46 ± 11.42	0.802
**Smoking, n (%)**	45 (30)	58 (38.66)	0.058
**Hypertension, n (%)**	97 (64.66)	96 (64)	0.500
**Familial history, n (%)**	43 (28.66)	40 (26.66)	0.398
**Anthropometric data**			
**Height (cm), mean ± SD**	169.63 ± 22.01	171.43 ± 19.38	0.001
**Weight (kg), mean ± SD**	76.75 ± 25.27	91.26 ± 41.34	0.001
**BMI (kg/m**^**2**^**), mean ± SD**	25.78 ± 5.97	28.78 ± 8.15	0.001
**WC (cm), mean ± SD**	99.17 ± 5.89	101.33 ± 5.19	0.803
**Laboratory data**			
**TG (mg/dl), mean ± SD**	128.01 ± 77.164	134.34 ± 25.43	0.480
**Chol (mg/dl), mean ± SD**	124.80 ± 59.66	128.91 ± 46.54	0.551
**LDL (mg/dl), mean ± SD**	97.82 ± 25.65	104.86 ± 31.69	0.324
**HDL (mg/dl), mean ± SD**	54.55 ± 10.90	46.43 ± 11.21	0.453
**FBS (mg/dl), mean ± SD**	110.34 ± 5.34	119.34 ± 6.43	0.860
**Leptin (ng/ml), (median (range))**	9.83 (7.99–11.76)	85.87 (82.20–88.37)	0.001
**Adiponectin (IU/ml), (median (range))**	28.68 (26.42–30.05)	8.65 (6.78–10.65)	0.001
**Leptin/adiponectin ratio, (median (range))**	0.51 (0.35–0.71)	7.45 (6.12–7.77)	0.001
**Angiographic data**			
**Vessel involvement, n (%)**	–		
**1 VD**	–	11 (7.33)	
**2 VD**	–	64 (42.66)	
**3 VD**	–	75 (50)	
**Gensini score (mean ± SD)**	–	23.62 ± 6.89	

*BMI* Body Mass Index, *WC* Waist Circumference, *TG* Triglyceride, *Chol* Cholesterol, *LDL* Low Density Lipoprotein, *HDL* High Density Lipoprotein, *FBS* Fasting Blood Sugar, *VD* Vessel Diseased

Logistic regression and multivariate analysis were applied for testing the predicting value of some plasma factors for CAD extension and severity. Since the difference between mean plasma leptin among cases and controls was high (85.87 ± 10.69 vs. 9.84 ± 5.36) with a significant difference (*p* = 0.0001), at the first step, the logistic identified only this factor as a powerful variable and other factors could not enter the equation. After deleting this variable three other factors were identified significantly (adiponectin, L/A ratio and waist circumference). Therefore, four most associated predictors for CAD extension were as follows: leptin, adiponectin, L/A ratio and waist circumference and the best predictor value for CAD extension in the current study was LEPTIN (Table 2).

**Table 2:** Bivariate relationship between different variables and serum leptin, adiponectin and L/A ratio in participants

Variable	Leptin (ng/ml)		Adiponectin (IU/ml)		Leptin/adiponectin	
	r	P	r	p	r	p
**Age (years**	−.04	.45	−.06	.32	.010	.86
**Hypertension, n (%)**	. 04	.49	.02	.69	−.004	.95
**FBS (mg/dl)**	.01	.86	−.05	.43	.04	.51
**TG (mg/dl)**	−.02	.72	.01	.91	.02	.79
**Chol (mg/dl)**	.02	.77	−.04	.49	.02	.78
**LDL (mg/dl)**	−.02	.71	−.04	.45	.02	.68
**HDL (mg/dl)**	.01	.83	−.05	.37	.06	.31
**Height (cm)**	−.09	.09	.16**	.007	−.10	.08
**Weight (kg)**	.04	.51	−.13*	.02	.06	.27
**BMI (kg/m**^**2**^**)**	.06	.33	−.13*	.02	.07	.26
**WC (cm)**	−.01	.83	−.09	.10	.04	.47
**Vessel involvement, n (%)**	.15**	.008	−.22**	.000	.18**	.001
**Stenosis (%)**	.08	.16	−.06	.30	.06	.30
**Gensini score (mean ± SD)**	−.01	.86	−.06	.33	.03	.64

*BMI* Body Mass Index, *WC* Waist Circumference, *TG* Triglyceride, *Chol* Cholesterol, *LDL* Low Density Lipoprotein, *HDL* High Density Lipoprotein, *FBS* Fasting Blood Sugar, *VD* Vessel Diseased

**Fig. 1:**
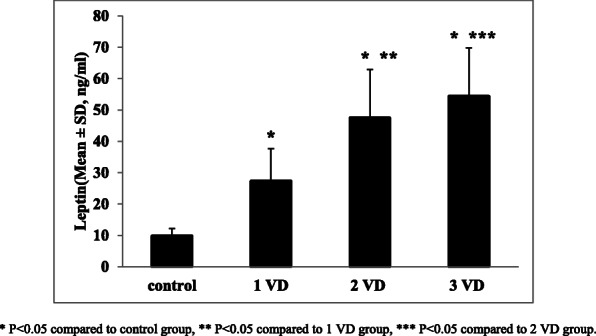
Mean ± SD of serum leptin level, based on the number of involved vessels (VD) in participants

The mean ± SD for the serum leptin level, based on the number of involved coronary vessels, is shown by Fig. 1. Based on the findings of this study, the mean serum leptin level in patients with one, two and three involved coronary vessels were 27.34 ± 10.32 ng/ml, 47.46 ± 15.43 ng/ml and 54.35 ± 15.43 ng/ml respectively and a positive and significant correlation between serum leptin levels and the number of coronary involved vessels was revealed (*p* = 0.008).

Also, logistic regression and t-test was applied for CAD extension and the results showed that those with 2 and 3 vessels involvement have 3.6 and 5.6 times higher chance of CAD than those with one vessel involvement respectively.

**Fig. 2:**
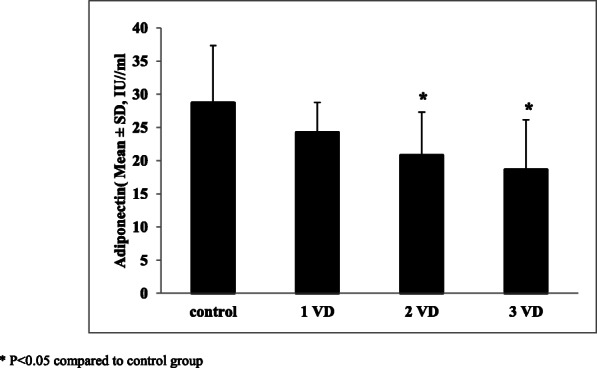
Mean ± SD of serum adiponectin level, based on the number of involved vessels (VD) in participants

The mean ± SD for the serum adiponectin level, based on the number of coronary involved vessels, is shown by Fig. 2. The mean ± SD serum adiponectin levels for one, two and three involved vessels were 24.24 ± 4.53 IU/ml, 20.78 ± 6.52 IU/ml, and 18.60 ± 7.54 IU/ml respectively and the correlation between the serum adiponectin levels and the number of involved coronary vessels was significant (*p* = 0.000).

**Fig. 3:**
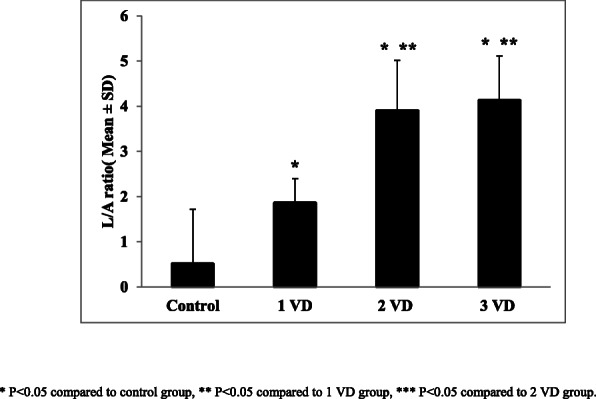
Mean ± SD of leptin/adiponectin ratio, based on the number of involved vessels (VD) in partcipants

The mean and standard deviation of L/A ratio, based on the number of involved coronary vessels is shown by Fig. 3. The mean serum L/A ratio among one, two and three involved coronary vessels were 1.84 ± 0.54, 3.90 ± 1.12 and 4.13 ± 0.98 respectively. There was also a significant correlation between the serum L/A ratio and the number of involved vessels (*p* = 0.001).

**Fig. 4:**
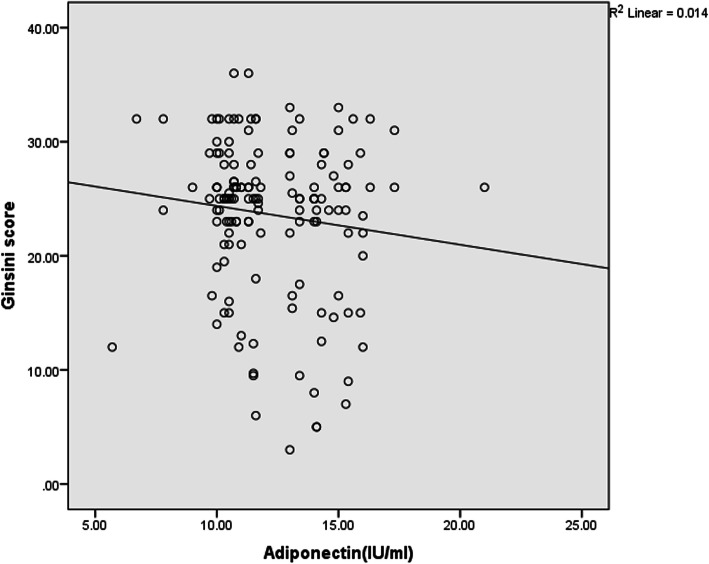
Correlations between serum adiponectin levels and Ginsini score in participants

The relationship between serum levels of adiponectin and the GS is shown by Fig. 4. According to the findings of this study, as the levels of adiponectin was increased, the GS was decreased, indicating an inverse association between serum adiponectin levels and the GS but this relationship was not significant.

**Fig. 5:**
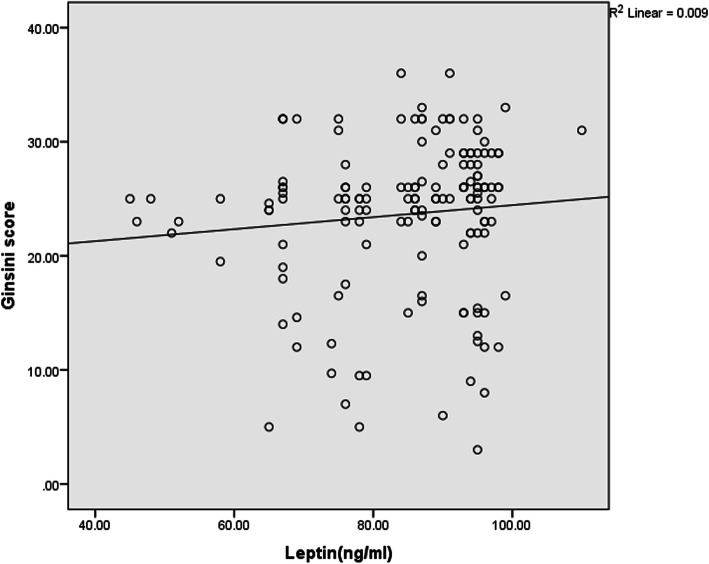
Correlations between serum leptin levels and Ginsini score in participants

The relationship between serum leptin levels and the GS is shown by Fig. 5 and based on the findings, the GS increased with increasing the serum levels of leptin, indicating a direct relationship between the serum leptin levels and GS insignificantly.

**Fig. 6:**
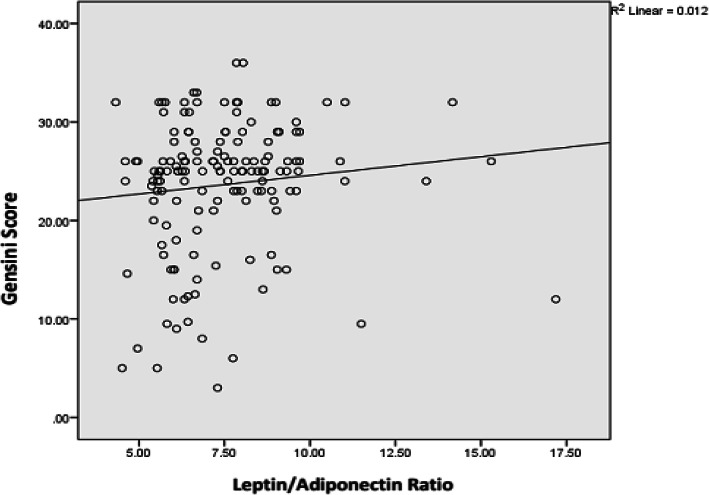
Correlations between leptin/adiponectin ratio and Gensini score in participants

Comparison between the serum L/A ratio and the GS also revealed an insignificant positive association and as the serum L/A ratio increased, the GS increased too (Fig. 6).

## Discussion

The current study investigated any relationship between the leptin, adiponectin and L/A ratio and the extent or severity of coronary artery disease based on the number of involved vessels and GS, and this was the first study in this field. In fact, several studies have reported the association between leptin or adiponectin and CAD separately; however, there is no study to show any correlation between the L/A ratio and angiographic findings. Our study findings showed that the serum adiponectin level among case group was significantly lower than that among healthy individuals. Additionally, a significant and inverse relationship between serum adiponectin levels and the extent of CAD, according to number of involved vessels, was revealed among patients. According to this finding, hypoadiponectinemia was identified as an independent risk factor for the extent of CAD and this finding was in accordance with previous reports [22]. Some studies have shown that hypoadiponectinemia was associated with CAD, particularly in the content of obesity, hyperinsulinemia, diabetes type 2, and dyslipidemia [23, 24]. Another study reported that hypoadiponectinemia could increase the risk of atherosclerosis and acute coronary syndrome [25]. Different mechanisms exist regarding the protective role of adiponectin in the control of atherosclerosis, but its anti-atherogenic role is not clear [25]; nevertheless, some studies reported that the anti-atherogenic effect of adiponectin was mediated through the inhibition of neointimal formation [26]. In vitro studies have shown a progressive neointimal damage and proliferation of smooth muscle in damaged arteries among mice without adiponectin [27, 28]. Furthermore, some studies have shown that the accumulation of adiponectin in the sub-endothelial spaces of damaged arteries causes an inhibition of genes related to inflammation, such as tumor necrosis factor-α (TNF-α), and markers related to endothelial malfunction, such as VCAM-1 and ICAM-1. With these effects, adiponectin may inhibit the adhesion of monocytes to the endothelial of cells, which is the first step in arteriogenesis, and their migration to the sub-endothelial spaces, as well as impose anti-atherogenic effects. The adiponectin-based inhibition of lipid accumulation in macrophages and increasing blood flow, resulted from vasodilator effects through nitric oxide (NO) production, are other beneficial effects of adiponectin in the inhibition of atherosclerosis [29, 30]. Our study showed that adiponectin has an inverse but insignificant relationship with the GS, namely an increase in the GS was associated to a reduction of adiponectin level among CAD patients. A study by Fukuta et al. reported that lower level of adiponectin was associated with LV diastolic dysfunction, among patients with CAD [31]. Another study reported that leptin was associated with increased atherogenic processes [32]. The results of our study indicated that serum levels of leptin were significantly higher among CAD patients than in healthy control subjects and leptin was also directly related to the extent of coronary artery disease, based on number of involved vessels. Unlike adiponectin, leptin significantly increased with increasing the numbers of involved vessels in CAD patients. Previous studies have shown a harmful effect of leptin in the pathogenesis of atherosclerosis and demonstrated that leptin was an independent risk factor for CAD [33]. It has been shown that leptin can increase the risk of CAD more than other risk factors for cardiovascular diseases such as sex, race, smoking, and body mass index [34]. A study reported that after excluding other risk factors, the odds ratio for increased disease severity among patients with high leptin levels was preserved [31]. The atherogenic mechanisms of leptin have not been clearly identified but its beneficial effects, such as increasing NO production, improving coronary blood flow [35] and its role in the process of angiogenesis have been reported [36]. On the other hand, some adverse effects of leptin have such as direct contact with the components of metabolic syndrome and impaired fibrinolysis have been reported [37]. Based on different reports, leptin can have other effects, such as obesity-related hypertension as well as pro-thrombotic properties, in the cardiovascular system [38–40]. Though the current study showed an higher significant serum level of Leptin among CAD patients than the healthy individuals, but it’s correlation with GS and CAD severity was not significant. The difference between our findings about relationship between leptin and severity of CAD with other reports is not clear but it may bay associated with the performance of coronary angiography and its interpretation by interventional cardiologists and score estimation or even a lower number of evaluated patients in the current study.

Based on the possible atherogenic, thrombotic and angiogenic effects of leptin, and existence of its receptors in the endothelial cells, rising of this factor in the blood may predict restenosis after some vascular accidents [41–43]. There is a controversy in the effects of leptin on CAD patients and though several study have reported a modest relationship between higher level of plasma leptin and risk of CAD incident [13] other have shown a protective effect for leptin [44, 45].

An important result of the current study was a significantly higher L/A ratio in patients with CAD in comparison with healthy individuals. This study also revealed that the L/A ratio was directly correlated with the extent but not severity of coronary artery disease. Additionally, the L/A ratio showed a direct correlation with the number of involved coronary vessels, indicating that patients with three-vessel coronary artery involvement had a higher serum L/A ratio than patients with two or one coronary artery involvement. A few studies have investigated the effects of serum L/A ratio on CAD. Kappelle and colleagues showed that the serum L/A ratio could be a useful marker for predicting the risk of the first cardiovascular event in men [46]. The advantage of our study was the investigation of serum L/A ratio in CAD patients compared with healthy subjects and its association with the numbers of involved coronary arteries and GS. Though the predictor effects of either serum leptin or adiponectin for the severity and extent of coronary artery disease has been reported previously [13], and our findings were almost in consistence with these reports; however, the current study revealed that the serum L/A ratio was another predictor rather than either leptin or adiponectin for CAD separately. This study was limited to compare the factors related to endothelial dysfunction or cardiac anatomic findings with the serum leptin, or adiponectin levels and/or the serum L/A ratio. A future cohort study, examining the relationship between the serum L/A ratio and patient’s prognosis would be helpful.

In the current study, serum levels of leptin were associated with incident of CAD and it was also directly related to the extent of coronary artery disease. As the number of involved vessels was higher, the serum level of leptin was higher too and this association could be shown as an increasing trend, so that leptin values in patients with one involved vessel compared to controls and those with two involved vessels compared to one and those with three involved vessels compared to two involved vessels were higher. A similar trend was revealed for the L/A ratio and the number of involved vessels in CAD patients but with an insignificant difference between 3 vessels compared to two vessels; however, in case of serum level of adiponectin, a negative relationship with involved vessels was appeared. Based on these results two concepts could be considered. Firstly, the serum level of both leption and L/A ratio are related to the increase in coronary atherosclerotic load. Secondly, at the start and during the progression of coronary atherosclerosis, the release of proaterogenic adipocytokines is increased, may be due to increased inflammatory process, and after a primary inflammation, this phenomenon declines and the serum level of leptin does not increase as markedly.

## Conclusion

As a conclusion, the effects of serum level of leptin or adiponectin were higher among CAD patients than healthy individuals and associated with the extent of coronary artery disease and our findings were in consistence with other previously reports. Also, the current study revealed that the serum level of leptin was a better predictor than either L/A ratio or adiponectin separately.

## Data Availability

Data are kept by the first authors and in case of need they are available by the corresponding author.

## Abbreviations

BMI: Body mass index
BP: Blood pressure
CAD: Coronary artery disease
CAC: Coronary artery calcification
Chol: Cholesterol
DBP: Diastolic blood pressure
FBS: Fasting blood sugar
GS: Gensini score
HDL: High density lipoprotein,
HTN: Hypertension
L/A: Leptin/adiponectin
LAD: Left anterior descending artery
LDL: Low density lipoprotein,
LCX: Left circumflex artery
MI: Myocardial infarction
NAFLD: Nonalcoholic fatty liver disease
NO: Nitric oxide
RCA: Right coronary artery
SBP: Systolic blood pressure
SD: Standard deviation
TG: Triglyceride
TNF-α: Tumor necrosis factor-α
VD: Vessel disease
WC: Waist circumference
